# Deciphering the modulation of gene expression by type I and II interferons combining 4sU-tagging, translational arrest and *in silico* promoter analysis

**DOI:** 10.1093/nar/gkt589

**Published:** 2013-07-05

**Authors:** Mirko Trilling, Nicolás Bellora, Andrzej J. Rutkowski, Miranda de Graaf, Paul Dickinson, Kevin Robertson, Olivia Prazeres da Costa, Peter Ghazal, Caroline C. Friedel, M. Mar Albà, Lars Dölken

**Affiliations:** ^1^Institute for Virology, University Hospital in Essen, University of Duisburg-Essen, Essen, D-45147, Germany, ^2^Computational Genomics Group, IMIM-UPF Research Programme on Biomedical Informatics, Barcelona Biomedical Research Park (PRBB), Barcelona 08003, Spain, ^3^Department of Medicine, University of Cambridge, Box 157, Addenbrooke’s Hospital, Cambridge CB2 0QQ, UK, ^4^Division of Pathway Medicine, University of Edinburgh Medical School, Edinburgh, EH16 4SB, Scotland, UK, ^5^SynthSys, University of Edinburgh, Edinburgh, EH9 3JU Scotland, UK, ^6^Institute of Medical Microbiology, Technical University Munich, Munich 81675, Germany, ^7^Institute for Informatics, Ludwig-Maximilians-University Munich, Munich 80333, Germany and ^8^Catalan Institution for Research and Advanced Studies (ICREA), Barcelona 08010, Spain

## Abstract

Interferons (IFN) play a pivotal role in innate immunity, orchestrating a cell-intrinsic anti-pathogenic state and stimulating adaptive immune responses. The complex interplay between the primary response to IFNs and its modulation by positive and negative feedback loops is incompletely understood. Here, we implement the combination of high-resolution gene-expression profiling of nascent RNA with translational inhibition of secondary feedback by cycloheximide. Unexpectedly, this approach revealed a prominent role of negative feedback mechanisms during the immediate (≤60 min) IFNα response. In contrast, a more complex picture involving both negative and positive feedback loops was observed on IFNγ treatment. IFNγ-induced repression of genes associated with regulation of gene expression, cellular development, apoptosis and cell growth resulted from cycloheximide-resistant primary IFNγ signalling. *In silico* promoter analysis revealed significant overrepresentation of SP1/SP3-binding sites and/or GC-rich stretches. Although signal transducer and activator of transcription 1 (STAT1)-binding sites were not overrepresented, repression was lost in absence of STAT1. Interestingly, basal expression of the majority of these IFNγ-repressed genes was dependent on STAT1 in IFN-naïve fibroblasts. Finally, IFNγ-mediated repression was also found to be evident in primary murine macrophages. IFN-repressed genes include negative regulators of innate and stress response, and their decrease may thus aid the establishment of a signalling perceptive milieu.

## INTRODUCTION

Interferons (IFNs) are soluble factors secreted on infection and capable of interfering (hence the name) with viral replication (1). IFNs lack direct intrinsic anti-viral capabilities and solely act as cytokines in an autocrine and paracrine manner initiating a global anti-infective change in the gene expression profile. IFNs induce many genes that are detrimental to cell survival and cell proliferation so that the expression of IFN-stimulated genes (ISGs) has to be prevented under ‘healthy’ or ‘uninfected’ conditions. On pathogen encounter, a robust defensive state is rapidly initiated to outpace microbial gene expression and replication. Owing to this binary nature, alternating between almost complete shut-off and rapid induction, IFNs have frequently been used as a model system to study stimulus-induced gene expression changes.

Human individuals with pathologic mutations within components of the IFN induction or signalling cascade suffer from recurrent episodes of infectious diseases elicited by opportunistic pathogens and frequently even fail to control live attenuated vaccine strains (2–6). Recombinant IFNs currently constitute the therapeutic backbone of the treatment of hepatitis B virus and hepatitis C virus infections. IFNs are subdivided into three classes: Type I IFN (IFNα/β), type II IFNs (IFNγ) and the recently identified type III IFNs (IFNλ), each being defined by a discrete receptor complex. Nevertheless, IFN-signalling cascades converge in common pathways and initiate gene expression almost exclusively via *janus kinase* (JAK)/*signal transducer and activator of transcription* (STAT)-signalling cascades (7). Type I IFNs (and type III IFNs) mainly induce the formation of STAT1:STAT2:IRF-9 heterotrimers [called *IFN-stimulated gene factor 3* (ISGF3)], whereas IFNγ mainly induces formation of STAT1 homodimers [called *gamma*-*activated factor* (GAF)]. To a lesser extent, IFNγ also induces formation of ISGF3 complexes (8,9) and IFNα/β also induces STAT1 homodimers (10). GAF and ISGF3 complexes translocate into the nucleus and bind to *gamma-activated sequences* (GAS) and *IFN-stimulated response elements* (ISRE), respectively, located in the vicinity of promoters of ISGs, to recruit the transcriptional machinery and facilitate gene expression. Consensus sequences of ISRE and GAS elements are significantly different and can thus be easily distinguished on DNA sequence level by bioinformatic means. Although subtle differences concerning the preference for certain sequence variations exist (11), basically all STAT family members bind to GAS elements (core motif TTCN_2-4_GAA). Therefore, it is not possible to deduce which STAT protein binds to a particular GAS element *in natura* based on its nucleotide sequence. ISRE elements closely resemble interferon regulatory factor (IRF)-binding sites (called IRF/E sites) confounding a sequence-based differentiation of ISRE and IRF/E sites (12,13).

IFNs themselves are subject to both positive and negative feedback loops. On encountering a pathogen, IFNβ [and IFNα4 in the mouse—but not in humans (14)] is expressed and signals through the JAK-STAT pathway to induce the expression of further IFNα subtypes, closing a self-amplifying loop (14,15). Additionally, components of the IFN signal transduction pathway, like STAT1 and STAT2, are themselves IFN inducible (16). Conversely, a number of IFN-inducible negative feedback mechanisms restrict IFN signal transduction to prevent an excessive and potentially detrimental hyper-activation of the innate immune system (e.g. auto-immunity). Known mediators of this counter regulation loop include *suppressors of cytokine signalling* (SOCS) (17,18), *ubiquitin specific peptidase 18* (19) and *protein inhibitors of activated STAT* proteins.

The currently accepted notion is that (especially on IFNβ encounter) the positive feedback constitutes an important and dominant event of IFN induction and signalling [see for example (20)]. It is assumed that the initial type I IFNs [IFNβ (and IFNα4 in the mouse)] stimulate the expression of gene products like the IFN-inducing transcription factor IRF7, components of the JAK-STAT signalling cascade (e.g. STAT1 and STAT2) and secondary IFNα subtypes to mount an effective innate immune response. Nevertheless, the regulation, temporal contribution and hierarchy of positive and negative feedback mechanisms to the IFN response are incompletely investigated and understood.

Standard profiling of total RNA for the detection of rapid changes in gene expression has a strong bias for detecting upregulation of short-lived transcripts (21). Therefore, the complex interaction network of positive and negative regulators within the initial phase of IFN signalling cannot be adequately studied by a sole assessment of transcript changes in total RNA. This is especially relevant for genes that are downregulated at the level of transcription, as their overall change in total RNA is critically defined by the intrinsic messenger RNA (mRNA) decay rate. This problem can be overcome by metabolic tagging, purification and analysis of newly transcribed RNA, using 4-thiouridine (4sU) (21,22). In this method, 4sU is incorporated in the newly transcribed RNA (4sU tagging). Following isolation of total RNA and thiol-specific biotinylation, newly transcribed RNA is separated from untagged pre-existing RNA using streptavidin-coated magnetic beads. All three RNA fractions (total RNA, labelled RNA and unlabelled RNA) are suitable for further analyses including quantitative PCR, microarray analysis (21,22) or next-generation sequencing (23–25).

**Figure 1.**
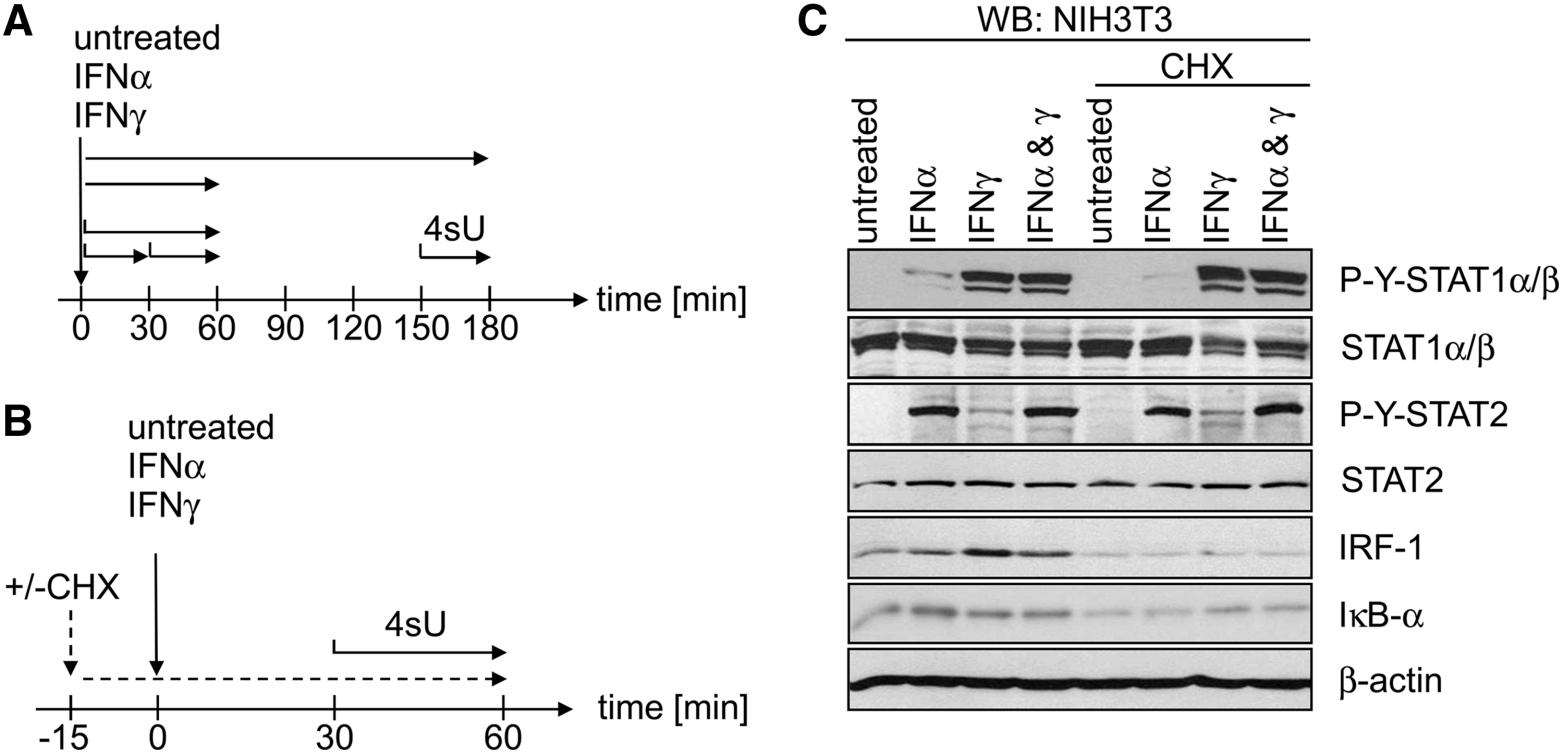
Experimental setup. (**A**) Schematic overview of the experimental setup to assess IFN responses by microarray analysis using newly transcribed and total RNA as published in (21). At different times of IFN treatment 4sU was added to cell culture medium for the indicated periods indicated with black horizontal arrows. Total RNA was prepared immediately after the end of labelling and newly transcribed RNA was purified and subjected to microarray analysis. (**B**) Schematic overview of the new experimental setup to test for the effect of translational inhibition using CHX or mock (DMSO) on IFN-mediated differential gene expression CHX. Fifteen minutes before begin of IFN treatment, CHX pre-treatment was started. Thirty minutes after begin of IFN treatment 4sU was added to cell culture medium to start RNA labelling. Thirty minutes later (=60 min of IFN treatment) total RNA was isolated and newly transcribed RNA was prepared. Three replicates of newly transcribed RNA from each of the six conditions were subjected to microarray analysis. (**C**) Immunoblot analysis of the signal transduction events in NIH-3T3 cells on incubation with IFNα (100 U/ml) and IFNγ (100 U/ml), respectively, in presence or absence of CHX (50 µg/ml). Cells were pre-treated for 15 min with CHX or DMSO (mock) and subsequently incubated for further 60 min with the indicated IFN. Cells were lysed and lysates were subjected to immunoblot analysis. Membranes were probed with the indicated antibodies.

In a previous study (21), we applied this approach to study changes in gene expression mediated by type I and II IFNs in murine fibroblasts. Changes in transcription rates were determined by Affymetrix arrays using newly transcribed RNA, labelled and purified following 0–30, 30–60 and 150–180 min of IFNα and IFNγ treatment (see Figure 1A for experimental setup). In newly transcribed RNA, induction of a large number of ISGs was already evident during the first 30 min of treatment. However, in subsequent experiments, we found important mediators of the positive feedback loop (e.g. IRF1) as well as of the negative feedback loop (e.g. SOCS3), to be substantially induced already within the first 15 min of IFN treatment. Thus, despite the ∼10-fold increase in sensitivity of our approach, primary (translation independent) effects could not be differentiated from secondary (translation dependent).

Our previous study identified a network of genes repressed by IFNγ showing strong associations with regulation of gene expression, cellular development, cell death, cellular growth and proliferation (21). Without knowledge of the underlying signal transduction mechanism, it had remained unclear whether this response represents a direct or an indirect effect of IFNγ treatment. To address these question and properly discriminate primary and secondary effects of type I and II IFNs, we now performed short-term 4sU tagging during IFN treatment in presence and absence of the translational inhibitor cycloheximide (CHX).

## MATERIALS AND METHODS

### Cell culture and 4sU tagging

Murine NIH-3T3 fibroblasts (ATCC CRL1658) and murine STAT1^-/-^ fibroblasts (26,27) were cultured in Dulbecco’s modified Eagle’s medium supplemented with 5% (v/v) new-born calf serum. For 30 min of metabolic tagging of newly transcribed RNA, 4sU (Sigma) was added to 500 µM final concentration into pre-warmed CO_2_-equilibrated medium. Cells were used only in between 5th and 15th passage after thawing; split twice weekly and 24 h before start of 4sU tagging. Total cellular RNA was prepared from cells using Trizol reagent (Invitrogen) following the protocol described by Chomczynski and co-workers (28). In the CHX experiments, NIH-3T3 cells were pre-treated with CHX (50 µg/ml) for 15 min before treatment with either mock, 100 U/ml IFNα or IFNγ for 60 min. During the last 30 min (=30–60 min of IFN treatment), 4sU was added at a final concentration of 500 µM to the cell culture medium (see Figure 1B). IFN activity and the effect of CHX treatment was checked in parallel using the ISRE Luc reporter cell line (9), which expresses luciferase under an IFN-inducible promoter (data not shown).

### Biotinylation and purification of 4sU-labelled RNA

Separation of total RNA into 4sU-tagged newly transcribed and untagged pre-existing RNA was performed as described (21). Briefly, biotinylation of 4sU-tagged RNA was performed using EZ-Link Biotin-HPDP (Pierce) dissolved in dimethylformamide at a concentration of 1 mg/ml. It was crucial to avoid dimethylformamide from getting in contact with incompatible plastic ware, as this may result in complete loss of newly transcribed RNA during the streptavidin purification. Biotinylation was carried out in 10 mM Tris (pH 7.4), 1 mM EDTA and 0.2 mg/ml Biotin-HPDP at a final RNA concentration of 100 ng/µl for 1.5 h at room temperature. Unbound Biotin-HPDP was removed by chloroform/isoamylalcohol (24:1) extraction using Phase-lock-gel (Heavy) tubes (Eppendorf). Afterwards, 1/10 volume of 5 M NaCl and an equal volume of isopropanol were added and RNA was precipitated at 20 000*g* for 20 min. The pellet was washed with an equal volume of 75% (v/v) ethanol and precipitated again at 20 000 *g* for 10 min. The pellet was re-suspended in 50–100 µl of RNase-free water. After denaturation of RNA samples at 65°C for 10 min followed by rapid cooling on ice for 5 min, biotinylated RNA was captured using µMACS streptavidin beads and columns (Miltenyi). Biotinylated RNA was incubated with 100 µl of µMACS streptavidin beads with rotation for 15 min at room temperature. The beads were transferred and magnetically fixed to the columns. Columns were washed three times with 1 ml of 65°C washing buffer [100mM Tris–HCl (pH 7.4), 10 mM EDTA, 1 M NaCl, 0.1% [vol/vol] Tween20] followed by three washes with room temperature washing buffer. Newly transcribed RNA was eluted from the beads by the addition of 100 µl of freshly prepared 100 mM dithiothreitol followed by a second elution round 5 min later. RNA was recovered using the RNeasy MinElute kit (Qiagen).

### Microarray sample labelling, hybridization and pre-processing

Total RNA (1.5 µg) or newly transcribed RNA (280 ng) was amplified and labelled using the Affymetrix One-Cycle Target Labelling Kit according to the manufacturer’s recommendations. Newly transcribed RNA samples were amplified and labelled according to the manufacturer’s protocol for mRNA. The amplified and fragmented biotinylated cRNA was hybridized to Affymetrix MG 430 2.0 arrays (mouse) using standard procedures. The complete microarray data set is available at Gene Expression Omnibus (GEO Series GSE30457).

### Microarray data processing and statistical analysis for analysis of IFN-mediated gene regulation

Data were processed and analysed using R and Bioconductor (29,30). Data analysis of total RNA (1 and 3 h of IFN treatment) and newly transcribed RNA samples (0–30, 30–60 and 150–180 min of IFN treatment) has been described in detail (21). The new microarray data on 30–60 min newly transcribed RNA (mock, IFNα, IFNγ; +/− CHX treatment) were processed independently of the recently published data but following a similar pipeline. Arrays were assessed for quality, ‘gene chip robust multiarray averaging’ (gc-RMA) normalized, filtered for low expression and analysed using an empirical Bayes moderated *t*-test.

‘Quality assessment’ consisted of RNA degradation plots, Affymetrix quality control metrics, sample cross-correlation, data distributions and probe-level visualisations.

‘Normalization’ incorporated background correction, normalization and probe-level summation by gc-RMA.

‘A non-specific filter’ in which all genes are retained that were called ‘present’ in at least one sample in the data set was applied before statistical testing (n = 27 759 probes passed this filter).

‘Statistical testing’ was performed by applying an empirical Bayes moderated *t*-test (31), which is the most robust test for small sample sizes. An increased rate of false-positive results owing to simultaneous testing on a large number of genes was corrected for by applying a multiple testing correction algorithm to the observed *P*-values, in this case using the Benjamini and Hochberg method (32). Finally, microarray data of all genes, showing significant differential gene expression (*P* < 0.01) in any condition of this and the previous experiment (21) were combined and used in the down-stream analyses. A schematic illustration of the data analysis workflow is provided as supplementary figure (Supplementary Figure S1).

### Cis-regulatory element analysis

For the bioinformatics analysis of DNA cis-regulatory elements, we first extracted all non-overlapping gene promoter sequences from the set of known mouse genes at UCSC Genome Browser (33). The promoter sequences used in the analysis spanned from −600 bp to + 100 bp relative to the annotated transcription start site (TSS). To predict the regulatory motifs, we used the complete collection of 639 vertebrate transcription factor-binding site (TFBS) position weight matrices (PWMs) from TRANSFAC (34), with thresholds PWM score ≥0.85 and PWM core score ≥0.99. Overlapping and redundant promoters were discarded, keeping one per gene, for each subset of regulated genes and the corresponding background. We then compared the distribution of frequencies of predicted motifs in the subset of genes of interest with the distribution in the rest of mouse genes using the non-parametric Mann–Whitney–Wilcoxon test. FDR correction were applied to *P*-values according to the Benjamini and Hochberg method (32). The enrichment, log_2_(d/b), were defined as the fold-ratio between the mean number of sites in the subset of promoters of regulated genes (d) and mean of the rest of mouse promoters (b). GC content and length of promoter region together with the information content of a motif influence the number of promoters with putative binding sites. The fraction of promoters for which sites are predicted and the densities per sequence are taking into account using our methodology. The procedure was performed using the R package (R Development Core Team, 2007) and an in-house C-program, previously used to detect enrichment of IRF sites in genes upregulated after nerve injury (35). Significant overrepresented motifs were defined as those in the subset of interest with a minimum 2-fold enrichment, a corrected *P* < 0.005 and present in at least five promoters and 25% of the promoters in the gene subset. In case of highly redundant TRANSFAC matrices (e.g. V$NKFB_Q6_01 and V$NFKAPPAB65_01), only the results obtained with one of the matrices, usually the one with the lowest *P*-value, are shown. The sequence logos were constructed *a posteriori* using the actual content of each data set.

### Immunoblot

Preparation of protein lysates, separation by SDS polyacrylamid gel electrophoresis (SDS–PAGE), blotting and detection by antibodies was carried out as described previously (36): Briefly, phosphate buffered saline washed cells were lysed in RIPA-buffer [50 mM Tris–HCl, 150 mM NaCl, 1% (vol/vol) IGEPAL, 1% Na-Deoxycholate (vol/vol), 0.1% (weight/vol) SDS, 1 mM dithiothreitol, 0.2 mM phenylmethylsulfonyl fluoride (PMSF), 1 µg/ml leupeptin, 1 µg/ml pepstatin, 50 mM NaF, 0.1 mM Na-vanadate with Complete protease inhibitors (Roche) (pH 7.5)]. Samples were normalized according to Bradford protein staining, and equal amounts were subjected to denaturing SDS–PAGE. Gels were blotted on nitrocellulose membranes (Schleicher and Schuell) and probed with indicated antibodies. The same membrane was used and consecutively stripped with reblot solution (Merck). The following commercially available antibodies were used: α-β-actin (Sigma-Aldrich); α-STAT1, α-Iκb-α and α-STAT2 (Santa Cruz); phospho-Tyr-STAT1 (Cell Signaling), IRF1 (Santa Cruz) and phosphor-Tyr-STAT2 (Millipore).

### nCounter design, measurements and data processing

To investigate the role of STAT1 on the network of IFNγ-repressed genes (IRepG), we chose 50 genes to be analysed by nCounter technology (see Supplementary Table S3 for complete list of genes). Details of the nCounter system are presented in full in reference (37). We hybridized 50–100 ng of RNA for 16 h with the code set and loaded into the nCounter Prep Station followed by quantification using the nCounter Digital Analyser. Code sets were designed and constructed to detect the 50 genes of interest. Each code set probe matches ∼100 bases long exonic sequence of the target genes (see Supplementary Table S4 for sequences). Background correction and normalization of data were performed as follows. For each sample, the average + 2 standard deviations (SDs) of background counts (negative controls) were calculated. For each gene of each sample, the average + 2 SDs were subtracted from the counts. Expression of the target genes was normalized by taking the geometric mean of the expression of the seven reference genes into account (38).

## RESULTS

### Combining 4sU tagging and translational arrest to dissect IFN-mediated transcriptional regulation

To properly differentiate primary from secondary effects and address the role of positive and negative feedback loops, we analysed IFN-induced changes in transcription rates in presence and absence of the translational inhibitor CHX. NIH-3T3 cells were pre-treated with CHX for 15 min (or DMSO as mock-treatment control) before starting the 60 min incubation with 100 U/ml of IFNα, IFNγ and mock, respectively. During the last 30 min of IFN treatment, 4sU was added to the cell culture medium to metabolically label newly transcribed RNA (see Figure 1B for experimental setup). Before performing microarray analysis, we validated the suitability of the experimental conditions. Immunoblot analyses were performed to monitor the effect of IFN and CHX on canonical IFN- and NF-κB-signalling at protein level. As expected, following 1 h treatment, STAT1 was found to be tyrosine phosphorylated on IFNγ treatment and, to a lesser extent, on IFNα treatment, whereas STAT2 phosphorylation showed the opposite regulation (Figure 1C). Ablation of translation by CHX before IFN addition did not prevent STAT phosphorylation. IκBα degradation, a hallmark and a prerequisite of canonical NF-κB activation, was not observed on IFN incubation but became weakly apparent after 75 min of CHX treatment. Induction of IRF-1 protein was more pronounced on IFNγ incubation compared with IFNα and was completely abrogated by CHX co-treatment. These results qualified the experimental setup as model system for the subsequent analysis of IFN signalling.

Following the experimental setup shown in Figure 1B, total cellular RNA from the six different experimental conditions was isolated, 4sU-tagged newly transcribed RNA was purified and subjected to microarray analysis using Affymetrix MG430 2.0 arrays (three biological replicates). Data were normalized using gc-RMA, and genes significantly regulated (>2-fold and *P* < 0.01) by either IFNα or IFNγ in presence or absence of CHX were identified. To include regulation of genes predominantly affected at later times of IFN treatment (at 3 h or 150–180 min), we also included the data from all genes identified in our previous study (21) for all subsequent analyses. This resulted in a list of 1031 probe sets significantly regulated by either IFNα or IFNγ (for complete list see Supplementary Table S1). An excellent correlation (Pearson correlation coefficient: IFNα: 0.84 and IFNγ: 0.87, respectively) for both IFNα- and IFNγ-mediated differential gene expression was observed in newly transcribed RNA at 30–60 min between the previously published and the new data set, thereby confirming the high reproducibility of this approach.

### Short-term CHX treatment affects the expression of a multitude of genes

**Figure 2.**
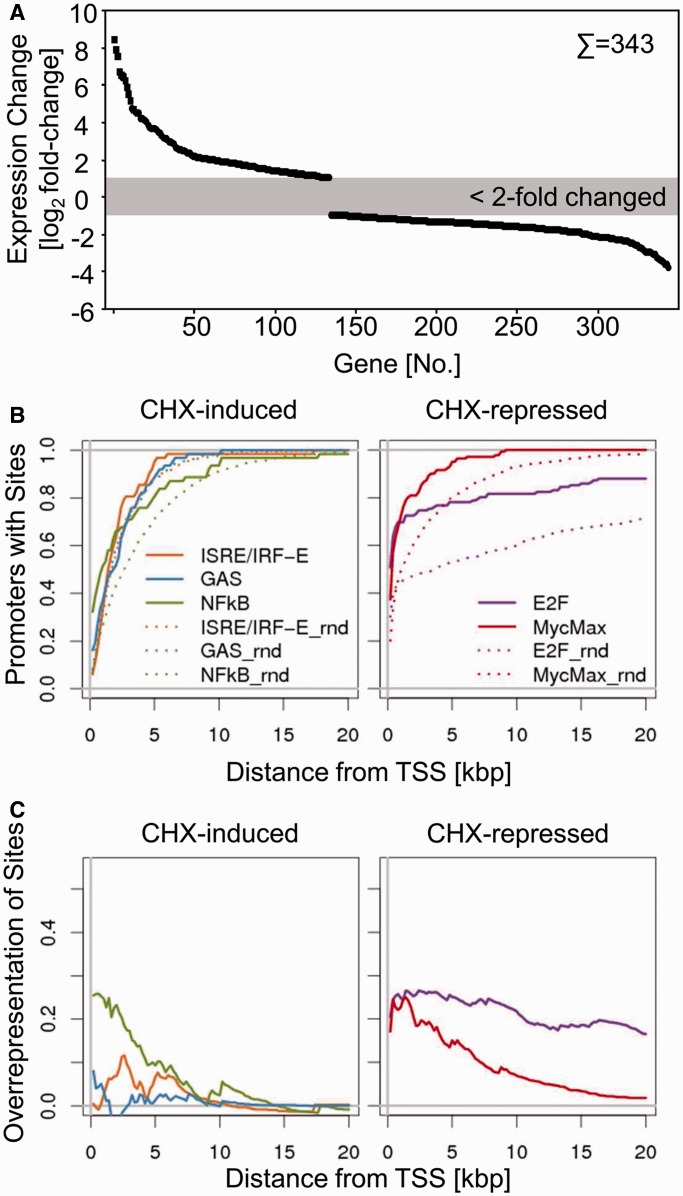
Changes in newly transcribed mRNAs upon inhibition of translation. (**A**) Expression change (depicted in a log_2_-scale) of significant changes of probe sets on treatment of CHX. (**B**, **C**) Distribution and enrichment of promoter/enhancer elements with respect to the TSS. The upper panel (**B**) depicts the cumulative percentage of genes harbouring the indicated element in comparison with randomly chosen gene set. The lower panel (**C**) depicts the enrichment in regulated genes in comparison with 1000 randomly chosen genes by subtracting from the curves shown in the upper panel.

Before having a closer look at the IFN-regulated genes, we first examined the effect of CHX treatment on cellular gene expression in the absence of IFNs. In total, 343 probe sets showed differential regulation (>2-fold and *P* < 0.01) on CHX treatment (Figure 2A; for complete list of genes see Supplementary Table S2). Significantly more probe sets showed down-regulation (n = 209) than upregulation (n = 134), consistent with a more prominent role of short-lived activatory regulators in maintaining steady-state transcription in comparison with inhibitory factors. The vast majority of CHX-induced changes detectable in newly transcribed RNA during 45–75 min CHX treatment were in the range of 2–20-fold. Interestingly, with the sole exception of ‘TNF receptor-associated factor 1’ (*traf1*) all transcripts showing >20-fold induction encode canonical histones. It is well described that CHX treatment results in significant induction of canonical histone mRNA levels owing to alterations in RNA synthesis, processing or decay (39–41). However, the extent and the celerity of this induction observed in newly transcribed RNA were not expected. The relative contributions of RNA synthesis, processing and decay can be dissected by gradually reducing the duration of labelling (25). By shortening the duration of 4sU labeling from 30 to as little as 5 min and analysing histone RNA expression by qRT-PCR, we show this regulation to be predominantly mediated by CHX-induced changes in RNA processing rather than changes in RNA synthesis (Supplementary Figure S2), which is consistent with previous studies.

### Cis-regulatory elements in CHX-regulated genes

Prolonged CHX treatment is known to result in NF-κB activation owing to decay of short-lived inhibitory proteins like Iκbα, which has an intrinsic protein half-life of 30–60 min (42).· To gain further insights into the transcription factors involved in CHX-mediated differential gene expression, we performed promoter analysis scanning for *cis*-regulatory elements in the proximal promoter regions of the corresponding genes. As the principle behind CHX-mediated induction of canonical histones has been shown to be histone mRNA specific and predominantly owing to their 3′-UTR resident stem-loop structures (43), we omitted these genes from further *in silico* analysis and focused on the remaining genes. We focused on DNA motifs significantly overrepresented between 600 bp upstream and 100 bp downstream of the TSS. We preformed promoter analyses using the TRANSFAC collection of TFBS profiles (34). Consistent with published data and with the reduction in IκBα observed in our immunoblot analysis (Figure 1C), this revealed a significant enrichment of NF-κB sites in genes over-expressed in the presence of CHX (33.9% of genes, *P* < 5.0 × 10^−^^8^, Table 1).

**Table 1.** Promoter analysis of CHX- and IFN-regulated genes

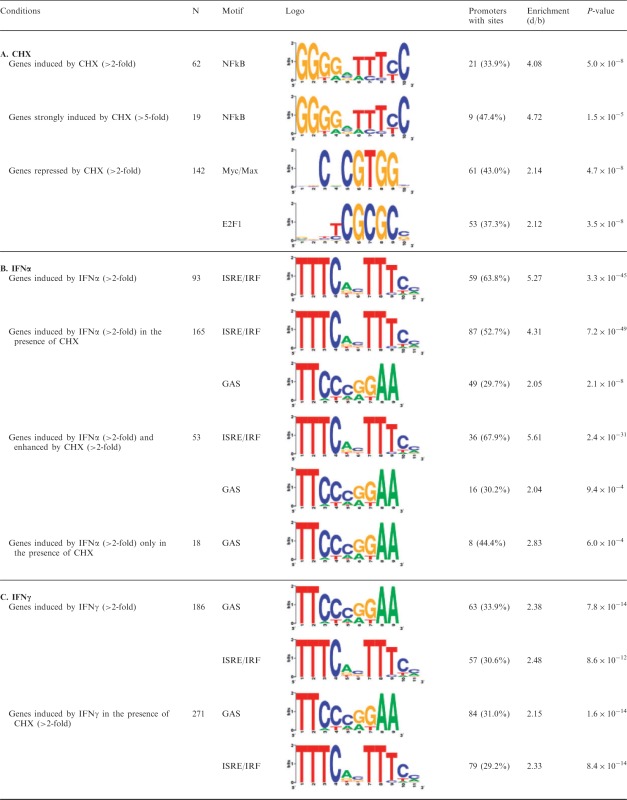
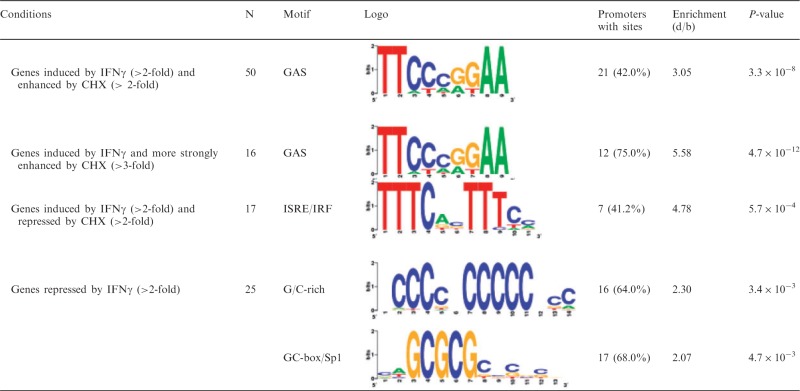

Over-represented *cis*-regulatory elements in genes showing differential expression in CHX (**A**), IFNα (**B**) and IFNγ (**C**)-treated cells. The number of genes that fulfilled the indicated conditions as well as the overrepresented transcription factor binding motifs (Logo), number of genes with sites (%), fold-ratio of mean number of sites in the data set and the mean in the background (d/b) and Mann–Whitney–Wilcoxon test corrected *P*-values are shown. Please note that some figures show differentially regulated probe sets, whereas this table depicts *bona fide* genes; therefore, the numbers necessarily vary. See Supplementary Table S5 for further details (e.g. d and b values).

Among the genes under-expressed in the presence of CHX, we found a significant enrichment of genes containing c-Myc/Max (43% of genes, *P* < 4.7 × 10^−^^8^) and E2F1 (37.3% of genes, *P* < 3.5 × 10^−^^8^) binding sites within their promoter/enhancer regions (Table 1). Both are considered to mainly fulfil transcriptional activator functions, although repressive effects have been described. c-Myc/Max is an essential heterodimeric transcription factor, composed of the two basic helix-loop-helix zipper proteins c-Myc and Max, which binds to E-boxes and regulates transcription to control cell proliferation, differentiation and cell death [reviewed in (44)]. E2F1 plays an important role in cell cycle control [reviewed in (45)]. In this context, it is noteworthy that c-Myc contains E2F-binding sites in its promoter (46,47), indicating that aforementioned CHX-affected gene sets are interrelated. Our data document the transcriptional changes elicited by CHX in absence of other stimuli owing to decay of transcriptional regulators known to have short protein half-lives, e.g. IkB-α, E2F and Myc (42,48–51).

To further substantiate the promoter/enhancer elements enriched among genes differentially expressed in presence of CHX, we calculated the cumulative abundance of NF-κB, E2F and Myc/Max consensus sites within 200 bp bins in the 20 kb upstream of the TSS of regulated genes in comparison with upstream regions of 1000 randomly chosen promoters. Owing to the focus of the present study on IFN-regulated genes, we also included ISRE/IRF and GAS consensus motifs, despite the fact that we did not observe a significant enrichment of these sites in CHX-regulated genes (Figure 2B and C). To highlight the relative enrichment, we plotted a subtraction of the presence in regulated genes versus the presence in a set of random genes (Figure 2C). Although the distribution of NF-κB sites showed a peak in the 2–5 kb region upstream of the TSS, the distribution of Myc/Max sites and especially of E2F sites is more flattened, suggesting that the effect of these transcription factors seems to be more far-reaching.

### Negative feedback loops already dominate secondary feedback mechanisms during the first hour of IFN treatment

**Figure 3.**
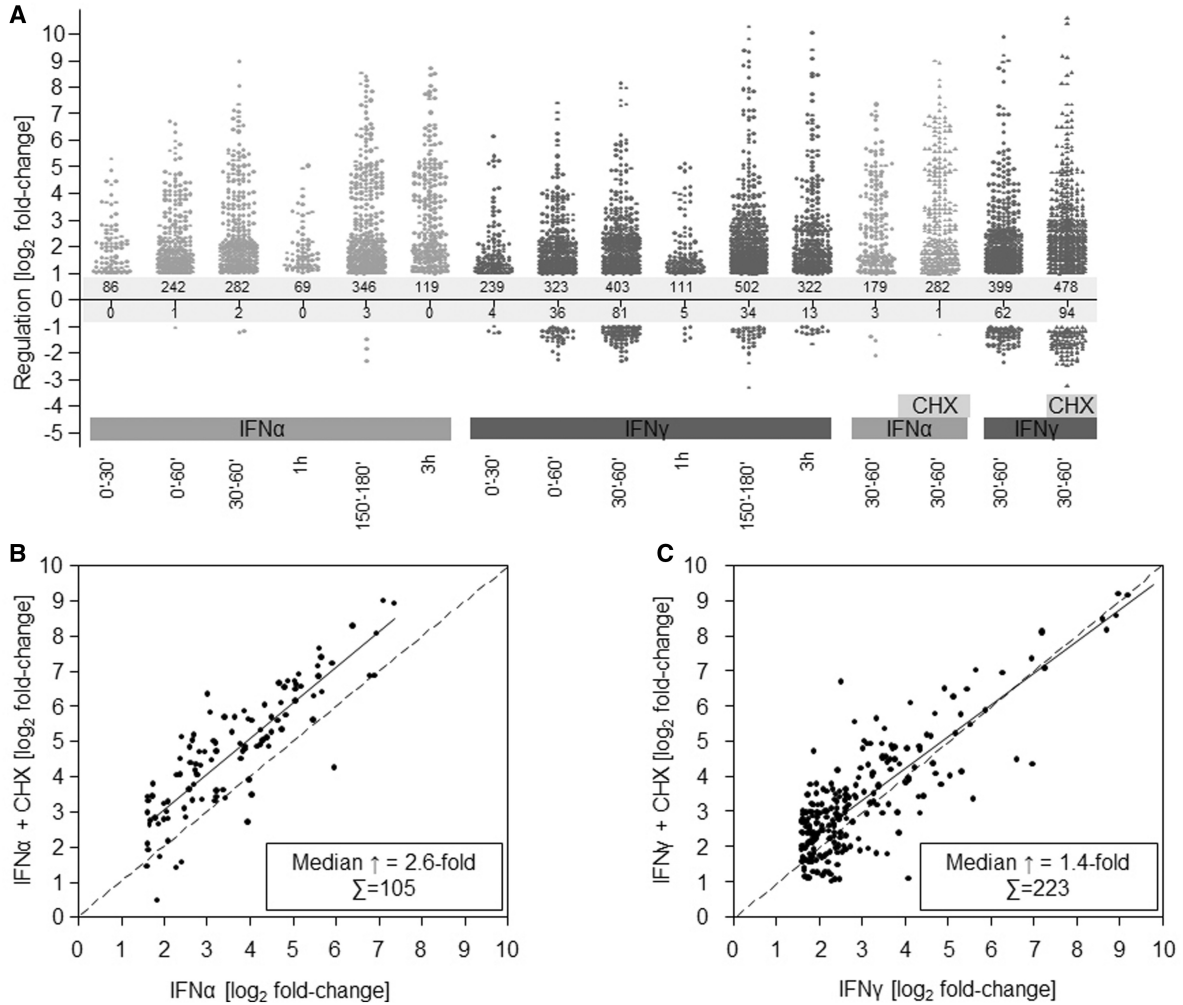
Negative feedback mechanisms dominate early IFN responses. (**A**) Differential regulation by IFNα (light grey) or IFNγ (dark grey) at indicated time points. Each significantly regulated probe set is represented by a dot. (**B**, **C**) Effect of CHX on IFN-mediated differential gene expression. The correlation of changes in gene expression in between genes regulated in absence (*x*-axis) or presence of CHX (*y*-axis) by either IFNα (**B**) or IFNγ (**C**) are depicted in a log_2_-scale. The insert displays the number of genes and the fold increase (median) owing to inhibition of translation.

At 30–60 min, IFNα and IFNγ significantly induced expression for 179 and 399 probe sets at least 2-fold, respectively (Figure 3A). CHX treatment increased the number of IFNα-induced probe sets from 179 to 282 and IFNγ from 399 to 478. In addition, it resulted in a ∼2.6-fold median enhancement of probe sets already induced >3-fold by IFNα in the absence of CHX. Interestingly, when we compared the fold-changes induced by IFNα in absence and presence of CHX, an almost parallel up-shift of the linear regression line became evident, indicating that almost all IFN-regulated probe sets are subjected to a similar expression increase on blocked translation (Figure 3B). This suggests a common negative feedback mechanism requiring *de novo* protein synthesis, which already dominates the secondary signalling events within 30–60 min of IFNα encounter. Thus, at least one essential component of this negative feedback mechanism requires active translation. Among the genes induced by IFNα in the respective time frame, we found a number of genes that have previously been implicated in the negative regulation of type I IFN responses, e.g. *usp18* (103.9-fold induced), *socs3* (4.59-fold induced), *socs2* (4.19-fold induced), *socs1* (2.39-fold induced) and *irf2* (1.72-fold induced).

For IFNγ-induced probe sets, a more complex picture was observed. Although CHX treatment also resulted in a noticeable, although less prominent, overall enhancement (∼1.4-fold) of IFNγ-induced changes, this was more heterogeneous than observed for IFNα and included both enhancement and inhibition indicative of partially opposing regulation circuits. Nevertheless, the net effect [19.8% more genes (399 versus 478) being significantly induced and a median ∼1.4-fold increase per gene in presence of CHX] underlines the importance of the translation-dependent negative feedback loop also for IFNγ responses (Figure 3C).

### Cis-regulatory elements within IFNα-induced genes

To gain further insights into the gene expression pathways responsible for the observed changes, we performed promoter analysis looking for *cis*-regulatory elements in the proximal promoter regions. We focused on DNA-sequences significantly overrepresented from 600 bp upstream to 100 bp downstream of the TSS. As expected from the wealth of knowledge concerning the IFN-signal transduction pathways, we observed ISRE/IRF sites (STAT2:STAT1:IRF-9 or IRFs) to be significantly overrepresented (*P* = 3.3 × 10^−^^45^) among IFNα-induced genes (n = 93, 63.8% of genes with sites, see Table 1). This was not significantly affected by co-incubation with CHX (52.7% of genes with sites, *P* = 7.2 × 10^−^^49^) consistent with ISRE/IRF-signalling representing the canonical and primary type I IFN signalling. On CHX co-treatment, GAS (STAT-binding) sites also became significantly overrepresented (29.7% of genes, *P* = 2.1 × 10^−^^8^). Most likely, binding of STAT1:STAT1 homodimers (termed AAF) or STAT1:STAT3 heterodimers is responsible for this regulation. In addition, both ISRE (67.9% of genes, *P* = 2.4 × 10^−^^31^) and GAS (30.2% of genes, *P* = 9.4 × 10^−^^4^) elements were found to be significantly overrepresented among genes, which were more strongly induced on co-incubation with CHX. This indicates that negative feedback not only shapes the quantity but also the quality of the IFNα response.

We also observed genes induced by IFNα only in presence of CHX but not by IFNα treatment alone. Interestingly, the vast majority of these were not induced even at 150–180 min of sole IFN treatment, indicating that this induction is not only due to a change in the kinetics of gene regulation but also a *bona fide* de-repression exerted by CHX treatment. Interestingly, GAS sites were significantly enriched (44.4% of genes, *P* = 6.0 × 10^−^^4^) among these genes, hinting at a translation-dependent control module acting on GAS-containing genes on encounter of IFNα which requires further studies.

### Cis-regulatory elements within IFNγ-induced genes

As expected, we found an enrichment (33.9% of genes, *P* = 7.8 × 10^−^^14^) of GAS sites within the proximal promoter regions of IFNγ-induced genes (Table 1). Similar percentages and *P*-values were found within genes upregulated by IFNγ in presence of CHX. Interestingly, we also observed significant overrepresentation of ISRE/IRF sites with comparable frequencies and *P*-values in the IFNγ-induced genes (30.6% of genes, *P* = 8.6 × 10^−^^12^), irrespective of CHX co-administration (29.2% of genes, *P* = 8.4 × 10^−^^14^), indicating that ISRE/IRF-containing genes constitute a considerable fraction of the IFNγ response. In addition, ∼20% of the genes induced by IFNγ, irrespective of the presence of CHX, harbour an ISRE/IRF element but no GAS site (within the assessed promoter/enhancer region), arguing against ‘hitchhiker’ effects as the sole explanation for this observation. This is consistent with previous data on STAT2 phosphorylation and ISGF3 activation in response to IFNγ (8,9,52). On the other hand, ISRE/IRF sites were found to be the only significantly overrepresented elements (41.2% of genes, *P* = 5.7 × 10^−^^4^) in the genes that showed reduced induction by IFNγ on CHX co-treatment. Therefore, the ISRE/IRF-response at 30–60 min of IFNγ treatment most likely constitutes a composite response, which both includes direct induction via ISGF3 (induced irrespective of CHX and repressed by translation dependent negative feedback mechanisms) as well as translation-dependent signalling mediated by, for example, IFNγ-induced IRF proteins like IRF-1 thereby at least partially explaining the more diverse effects of CHX on the IFNγ-induced genes.

### Temporal changes and stringency in occupancy of cis-regulatory elements

**Figure 4.**
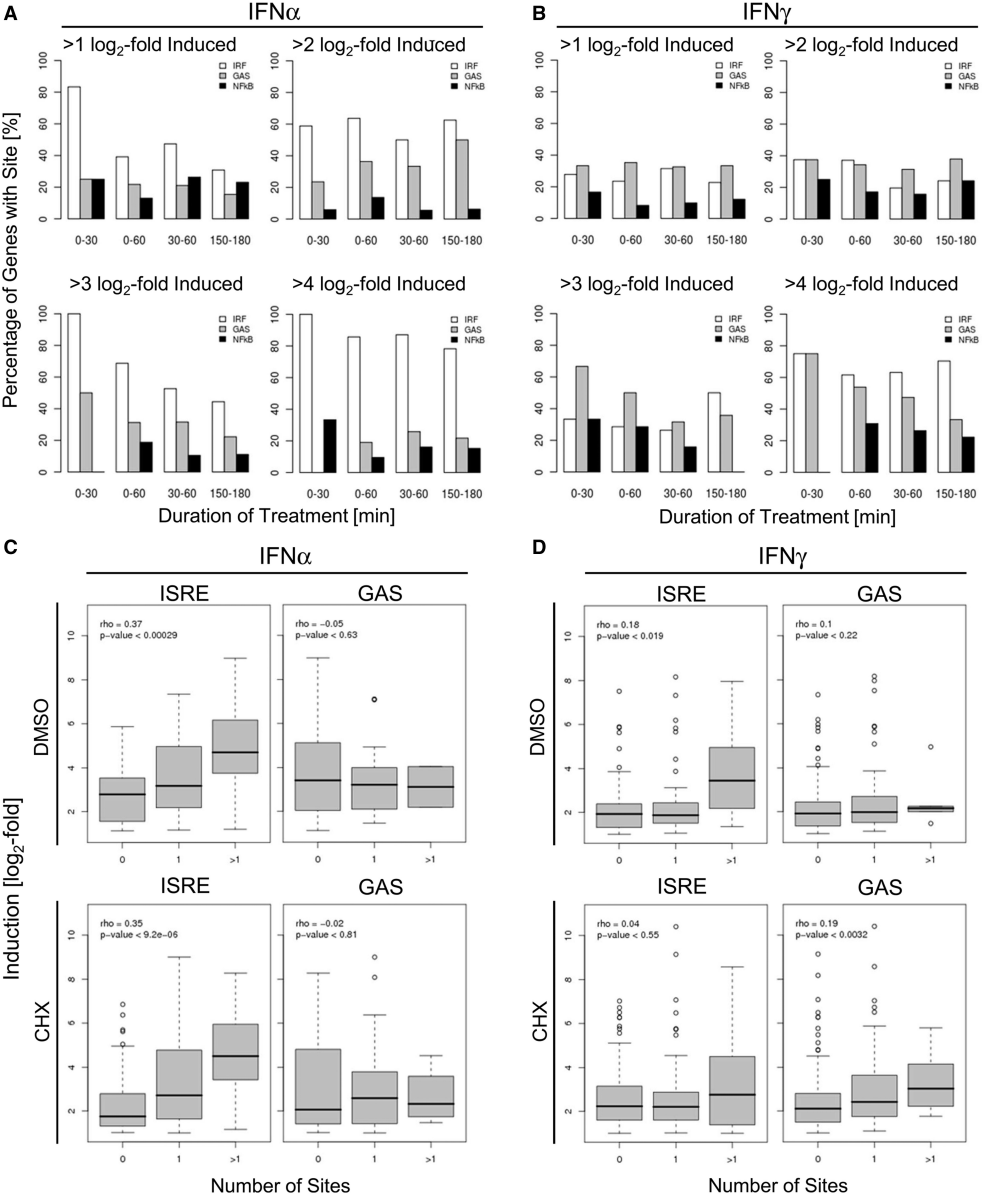
Temporal resolution (**A**, **B**) and co-operative nature (**C**, **D**) of TFBS enrichment in relation to transcriptional changes. (A) Percentage of genes harbouring an ISRE/IRF (white bars), a GAS (grey bars) or an NF-κB (black bars) element on IFN incubation within the indicated time frame (0–30, 0–60, 30–60 and 150–180 min of IFNα incubation, respectively) in respect to the individual strength of induction (more than two-, four-, eight- and 16-fold). (**B**) The same figure as in A, but for IFNγ. The median fold induction (depicted in a log_2_-scale) for IFNα- (**C**) and IFNγ-induced genes (**D**) is shown in respect to the number of ISRE/IRF or GAS elements in their promoter/enhancer (0, 1 or >1) on IFN incubation in absence (upper panel) or presence (lower panel) of CHX. The *P*-value of the correlation between number of sites and induction is indicated in the diagrams.

We assessed the frequency of genes with a given induction strength (2^1 ^-, 2^2 ^-, 2^3 ^- or 2^4^-fold induced) at a given time interval post IFN treatment containing either ISRE or GAS sites (Figure 4A and B). Although we did not find a significant enrichment of NF-κB motifs within ISGs, we included NF-κB as control owing to its prominent role in innate immune signalling and its previously described implication in IFN signal transduction (53). When looking at genes induced >2-fold by IFNα, we found, irrespective of the labelling interval, 60% of genes to harbour ISRE/IRF sites. Only 20–30% of genes contained GAS sites and <20% contained NF-κB sites (Figure 4A). The stronger genes were induced by IFNα within the first 30 min, the higher was the likelihood to find ISRE/IRF sites, culminating in the finding that 100% of genes induced >8-fold within the first 30 min contained ISRE/IRF elements (Figure 4A). This indicates that strong and rapid induction by IFNα can be sufficiently explained by the presence of ISRE/IRF sites within the proximal promoter/enhancer region.

In >2-fold IFNγ-induced genes, we found enrichment of ISRE/IRF and GAS sites with similar percentages (30–40%) (Figure 4B). These percentages increased to 70–80% in genes induced more vigorously by IFNγ (>8 - or even >16-fold) within the first 30 min of IFNγ treatment but did not reach 100% as observed for the most strongly IFNα-induced genes (Figure 4B). Interestingly, strongly IFNγ-induced genes showed a motif changeover during on-going IFNγ treatment with the percentage of genes harbouring a GAS site gradually decreasing from >70% (0–30 min) to <33% (150–180 min). In contrast, the percentage of genes with an ISRE/IRF site slightly increased from 50 to 65%. This suggests a temporal change in the transcriptional program during IFNγ treatment from an immediate-early GAS-response towards a secondary ISRE/IRF-mediated response later on, likely due to the second wave of regulation mediated by transcription factors like IRF-1.

### Cooperative effects of ISRE/IRF and GAS sites

When we divided genes in classes depending on the number of ISRE/IRF elements, we observed that genes harbouring an ISRE/IRF element in their proximal promoter regions are more strongly induced upon IFNα than those lacking an ISRE/IRF element and that genes harbouring more than one ISRE/IRF site are also more strongly induced by IFNα compared with genes with only one element (Figure 4C; *P* < 0.00029). The same was observed for the genes induced by IFNα in presence of CHX; *P* = 9.2 × 10^−^^6^). This suggests an additive and cooperative nature of ISRE/IRF-E elements. A biochemical counterpart to this observation has been made previously (54). Among IFNγ-induced genes, we also observed an increased induction of genes harbouring more than one ISRE/IRF site within their proximal promoter region (Figure 4D)—although this was less pronounced in terms of significance (*P* < 0.019) and intensity. GAS sites within IFNα-induced genes did not show such a trend. However, the number of IFNα-inducible genes containing more than one GAS site was only low (n = 2).

To exclude the possibility that regulation via ISRE/IRF sites might simply mask cooperative effects of GAS sites, we had a closer look at IFNγ-inducible genes, which lack ISRE/IRF sites but harbour no, one, or more than one GAS site within their promoter/enhancer elements, respectively. Interestingly, we found that GAS sites seemingly act cooperatively on IFNγ and CHX co-treatment but not on IFNγ-treatment alone (Supplementary Figure S3), which might indicate that negative feedback-loops mask such additive effects on promoters containing multiple GAS sites.

### Cis-regulatory elements in distal promoter regions

**Figure 5.**
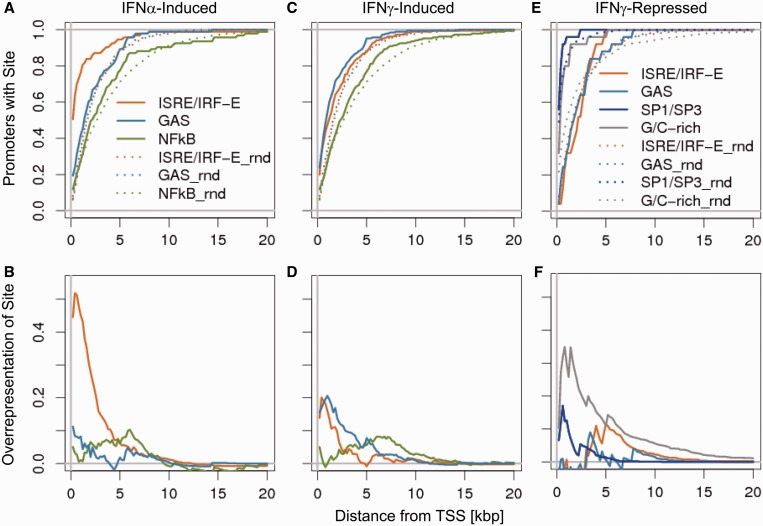
Positional distribution of TFBS. Distribution and enrichment of elements [ISRE/IRF (orange), GAS (light blue), NF-κB (green), SP1/SP3 (dark blue) and G/C-rich (grey)] in respect to the TSS for IFNα-induced (**A**, **B**), IFNγ-induced (**C**, **D**) and IFNγ-repressed (**E**, **F**) genes. Curves derived from regulated genes are shown as straight lines and the non-regulated control genes are depicted as dotted lines. The Upper panel (**A**, **C**, **E**) shows the additive percentage of genes harbouring the indicated element in comparison with randomly chosen gene set. The lower panel (**B**, **D**, **F**) depicts the over-representation of elements in the regulated genes in comparison to the non-regulated genes by subtracting of the curves shown in the upper panel.

Although the majority (>70%) of IFNα-induced changes can likely be attributed to ISRE/IRF and GAS sites present in proximal promoter regions (PPR, −600 to +100 bp from TSS) about half of the IFNγ-induced genes had no ISRE/IRF, GAS or NF-κB sites in their PPR. As we did not find any additional significantly overrepresented motif available in the TRANSFAC database in the PPR, we extended our promoter analysis to look for ISRE/IRF, GAS or NF-κB sites as far as 20 kb upstream of the TSS. The proportion of promoters that had at least one of the motifs at a given distance for IFNα- and IFNγ-induced genes compared with non-induced genes is depicted in Figure 5. After subtracting the background signal, it can be clearly appreciated that the strongest signal for ISRE and GAS is located in the PPR. Compared with promoters of IFNα-induced genes, promoters of IFNγ-induced genes showed a less pronounced accumulation of ISRE motifs but a stronger accumulation of GAS sites.

### The network of IRepG constitutes a primary IFN response

**Figure 6.**
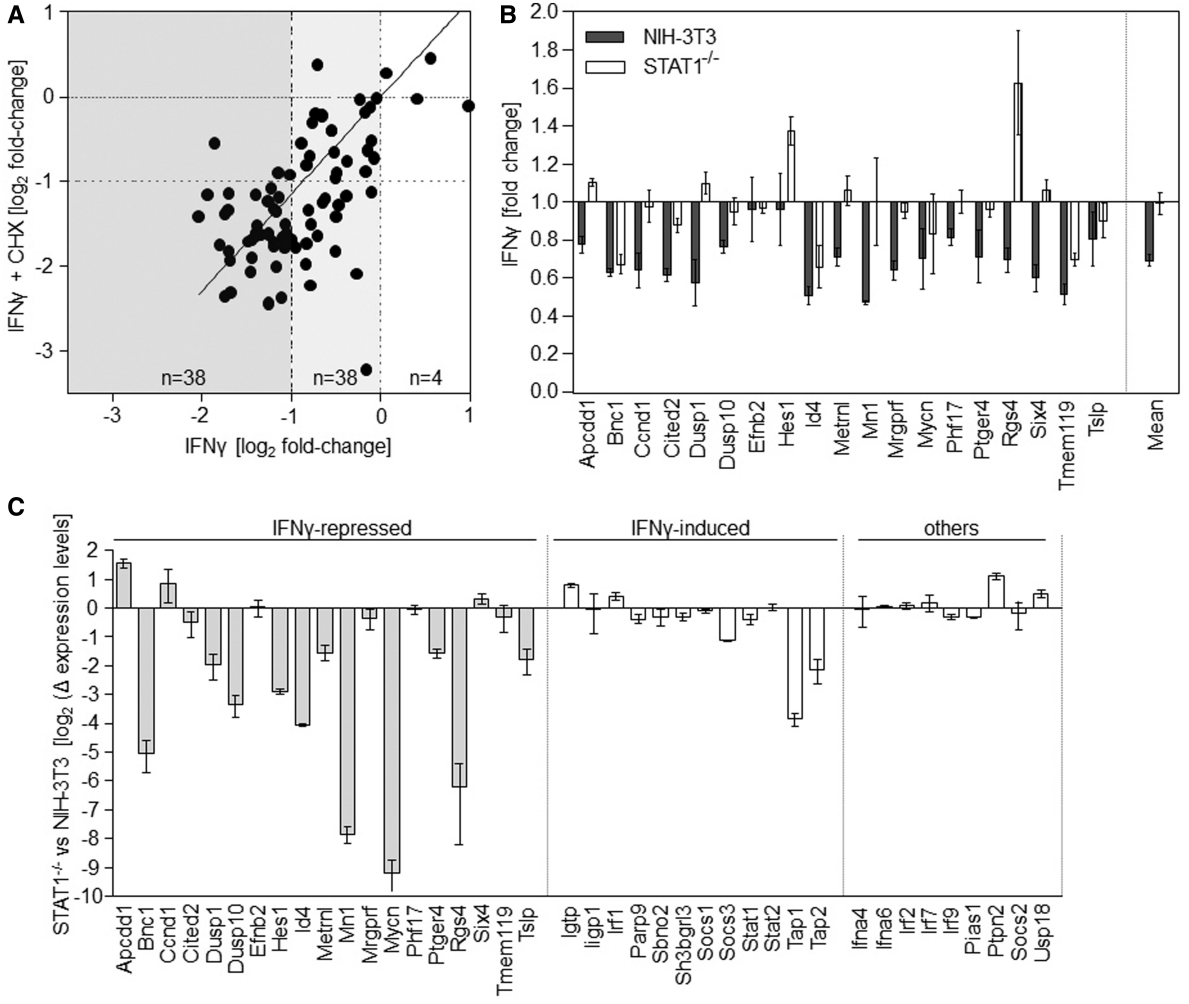
IFNγ-mediated gene repression depends on STAT1. (**A**) Comparison of genes repressed by IFNγ (30–60 min) in presence (*y*-axis) and absence (*x*-axis) of CHX. The number of regulated genes within the rectangles illustrated in different grey scales are indicated. (**B**, **C**) nCounter analysis of the effect of IFNγ on 50 marker genes in NIH-3T3 and STAT1^−/−^ fibroblasts. Cells were treated with 100 U/ml of IFNγ or mock for 60 min. In all, 500 µM 4sU was added from 30 to 60 min of treatment. Newly transcribed RNA was purified and subjected to nCounter measurements for transcripts of 50 selected genes. Data were normalized based on seven house-keeping genes showing stable expression levels in both cell lines. For 19 IRepGs (**B**), combined data from two independent experiments, consisting of two biological replicates are shown. Although expression of 17/19 IRepGs was observed in NIH-3T3, only 4/19 genes showed >20% reduced expression levels in the STAT1^−/−^ cells (Fisher’s exact test: *P* < 0.0001). (**C**) Dependency of gene expression on STAT1 in IFNγ-naïve cells. nCounter measurements of basal expression levels in NIH-3T3 and STAT1^−/−^ fibroblasts are shown. Expression of 11/19 (58%) of the IRepGs but only two of the 21 other genes (9%) was dependent on STAT1 (Fisher’s exact test: *P* = 0.0019).

As stated earlier in the text, IFNγ is capable to repress the expression of a set of genes (21). We were therefore interested to test whether this response constitutes a primary or secondary response to IFNγ. We found the majority of the ∼80 probe sets described to be repressed by IFNγ to be also >1.5-fold downregulated on IFNγ treatment in our current experiments (Figure6A). Most importantly, 35 of 38 probe sets, which showed >2-fold repression in both the old and new experiment by IFNγ treatment alone, were also >2-fold repressed by IFNγ in presence of CHX. This finding excludes the necessity of *de novo* protein translation for this transcriptional repression and reveals a direct role of IFNγ-signalling in transcriptional repression. Therefore, this gene family comprises genuine IRepGs. Expression of these probe sets was not generally repressed by CHX treatment alone (median regulation by CHX alone = 1.2-fold upregulation). Most interestingly, the number of significantly repressed probe sets even increased from 62 to 94 on CHX addition, suggesting that IFN-induced gene repression is also under the control of a rapid translation-dependent negative feedback mechanism.

### Cis-regulatory elements within IRepGs

IRepGs did not show any significant enrichment of GAS or ISRE/IRF sites. Nevertheless, these gene-repressions even occurred in presence of CHX, thus constituting a *bona fide* primary IFNγ response. The absence of GAS and ISRE elements in their proximal promoter regions is consistent with the well-established role of ISGF3 and GAF in induction but not suppression of transcription. Interestingly, we found a significant overrepresentation (64.0% of genes, *P* < 3.4 × 10^−^^3^) of G/C-rich promoter/enhancer elements and corresponding SP1/SP3-DNA binding sites, so called GC-boxes (68.0% of genes, *P* < 4.7 × 10^−^^3^, see Table 1). We observed a gradual enrichment of SP1/SP3 sites and even more frequent of G/C-rich stretches near the TSS (Figure 5E and F).

For an individual IRepG (‘lipoprotein lipase’), negative regulation by SP1 and SP3 has been described (55). Our data imply that such regulation is more widespread than commonly accepted and thus deserves a thorough investigation concerning the underlying receptor-proximal events and contribution to antiviral, anti-pathogenic and anti-tumorigenic IFNγ functions.

### IFNγ-mediated repression depends on STAT1

Although the prominent role of STAT1 for IFNγ is well established, disagreement exists concerning the relevance of STAT1 for IFNγ-induced gene repression (56,57). To clarify this issue, we used STAT1-deficient fibroblasts (26) and used Nanostring nCounter technology for accurate multiplex measurements of transcripts of 50 genes (19 IRepGs, 10 house-keeping genes, 12 IFNγ-induced genes as well as 9 gene products involved in canonical IFN-signalling, see Supplementary Table S3 for complete list of genes). Both STAT1-expressing and STAT1-deficient fibroblasts were treated with either IFNγ or mock for 60 min as described in Figure 1B and newly transcribed RNA was purified. RNA samples derived from two independent experiments (two biological replicates/experiment) were analysed. Data were normalized according to the seven most stable house-keeping genes. Two genes (*ifna4* and *ifna6*) were not detectable by the NCounter probes and thus excluded from the analysis.

In agreement with the central role of STAT1 for IFNγ signal transduction, strong induction of the 12 IFNγ-inducible genes was observed in normal but not in STAT1^−^^/^^−^ fibroblasts (see Supplementary Figure S4). Consistent with our previous data, IFNγ treatment resulted in significantly reduced transcript levels for 17/19 (89%) of the IRepGs in STAT1-expressing fibroblasts (Figure 6B). Interestingly, this repression was not detectable in the STAT1-deficient fibroblasts where only three genes showed >20% repression (Fisher’s exact test: *P* < 0.0001). Although the IFNγ-induced repression was less prominent than observed by microarrays, these data reveal IFNγ-mediated repression to depend on STAT1. Next, we compared expression levels of the 48 genes in IFN-naïve cells (Figure 6C). Interestingly, basal expression levels of 11/19 (58%) IRepGs were strongly dependent on the presence of STAT1, whereas STAT1 was only required for 2/21 (9%) of the class of IFN-induced genes in absence of IFNγ (Fisher’s exact test: *P* = 0.0019), namely, *tap1* and *tap2*. These data indicate a previously unsuspected role of alternative STAT1 complexes in maintaining basal expression levels of IRepGs requiring further studies.

### IFNγ-mediated gene repression occurs in murine bone marrow-derived primary macrophages

**Figure 7.**
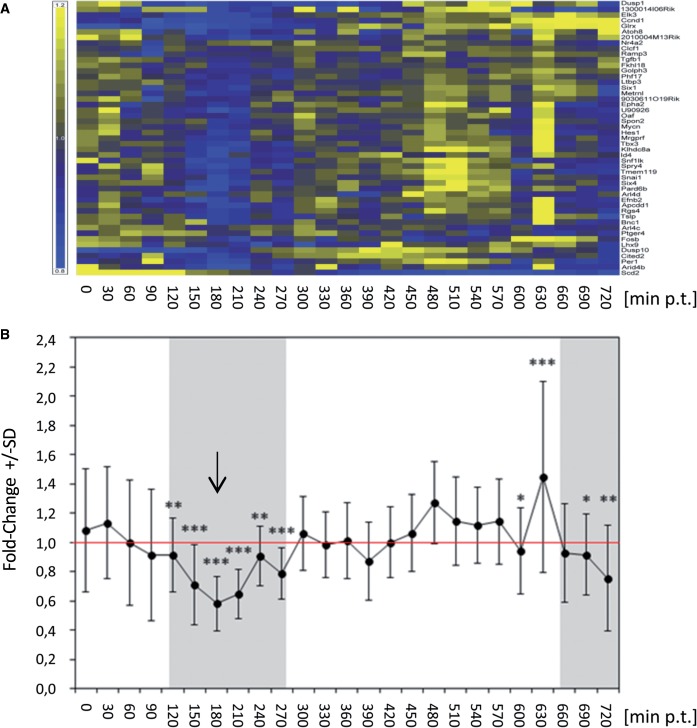
Transient down regulation of IRepGs in primary mouse bone marrow derived macrophages. In a recently published study (58), mouse BMDM were treated with 10 U/ml IFNγ. Cells were lysed with Trizol every 30 min during the first 12 h of treatment and the obtained RNA samples were hybridized to Mouse Agilent V2 arrays. Gene probes matching to the list of IRepGs we identified in murine fibroblasts were identified and ‘per gene normalized’ values were calculated from log_2_ expression values. (**A**) Heat map of ‘per gene normalized’ log_2_ expression values over the 12 h time course post-treatment (p.t.) are shown. Gene probes were clustered by Euclidean distance (Yellow—upregulated, Blue—downregulated). (**B**) Line graph of mean per gene normalized log2 expression values. The arrow indicates the time point showing the strongest down regulation.

IRepGs are characterized by a shorter median RNA half-life (median t_½_ = 90 min) than mouse transcripts in general (median t_½_ = 295 min) (21). Based on this short half-life, we hypothesized that their regulation should become detectable in total RNA. To assess the conservation and extent of regulation of IRepGs in another cell type considered more relevant to innate immunity, we took advantage of a comprehensive set of data analysing IFNγ-induced changes in total RNA levels in murine bone marrow-derived primary macrophages (BMDMs) obtained by the Ghazal laboratory (58). In this data set, IFNγ-induced changes in mRNA expression levels were determined in 30 min intervals for a 12 h period using Mouse Agilent V2 arrays. Interestingly, the vast majority of IRepGs, we identified in fibroblasts also showed significant downregulation in BMDMs between 150 and 210 min post-treatment (Figure 7). Therefore, regulation of IRepGs is not restricted to fibroblasts. The temporal delay highlights the difference in sensitivity for analysing short-term changes in gene expression in newly transcribed compared with total cellular RNA. This provides strong evidence that the respective RNA transcripts are also short-lived in primary murine macrophages. In addition, their regulation appears to be subject to similar negative feedback regulation as observed in fibroblasts. In conclusion, IRepGs represent a group of genes with regulatory functions involved in regulation of gene expression, cellular development, cell death and cellular growth and proliferation transiently repressed during the first few hours of the IFNγ response.

## DISCUSSION

In this study, we combined transcriptomics of nascent RNA (4sU tagging) with short-term translational inhibition using CHX to study the kinetics and molecular mechanisms of IFN-mediated differential gene expression. Inhibition of protein synthesis using CHX is a commonly used approach to dissect translation-dependent from translation-independent changes in gene expression. As changes in gene expression following a stimulus generally take ∼3–6 h to become apparent in total RNA for the majority of genes, prolonged translational arrest is required to elucidate the nature of the observed changes. 4sU tagging provides quantitative data on the kinetics of transcriptional changes in a time scale of ≤30 min (21), thereby detailing the real-time kinetics of transcription factors activity. So far, the power of the two approaches has never been combined. In the present study, we used 4sU tagging, short-term CHX-treatment and *in silico* promoter analysis to depict the real-time contribution of feedback mechanisms during the first hour of the response of fibroblasts to type I and II IFNs.

Interestingly, differential gene expression caused by both IFNα and IFNγ treatment was significantly enhanced on ablation of translation by CHX, revealing a dominant global role of negative feedback loops already within the first hour of treatment. Of note, although CHX treatment resulted in a uniform enhancement of the IFNα-induced genes by ∼3-fold, its effect on IFNγ-regulated was more diverse, indicating a greater contribution of positive feedback loops, e.g. mediated by induction of IRF-1, during the first hour of IFNγ treatment. Translation-dependent negative feedback loops dominating early IFN responses have been described for individual genes [ISG54 (59), IFN-IND1 and IFN-IND2 (60)] in human melanoma cells and human diploid fibroblasts. Nevertheless, it will be important to extend this kind of analysis to other cell types and to different species (especially humans) to evaluate whether the dominance of rapid negative feedback regulation constitutes a general feature of the IFN response.

Positive feedback loops may well play a more important role in immunologically more active cells, e.g. macrophages or dendritic cells. For individual genes, enhanced induction on CHX co-treatment has been reported. As such, CHX ‘supra-induces’ *ISG54* mRNA amounts (59) and increases mRNA levels of *IFN-IND1* and *IFN-IND2* (60). However, the well-known anti-proliferative and even pro-apoptotic nature of IFNs suggests that rapidly activated inhibitory regulative circuit alleviate detrimental effects of IFN-regulated genes. This is exemplified by the fatal effect of IFNγ in SOCS1-deficient mice (61). Nevertheless, it is tempting to speculate that (some) cells might be equipped to de-repress this rapid negative regulation under particular situations, e.g. when additional inflammatory stimuli (e.g. toll-like receptor, NOD-receptor, RIG-like receptor co-engagement or TNF-stimulation) are encountered.

Among IFN-induced genes, we found a significant enrichment of ISRE/IRF consensus elements and GAS sites. As expected, IFNα-induced genes were characterized by the presence of ISRE/IRF elements. On co-administration of CHX and IFNα, GAS sites become more strongly enriched, suggesting that a translation-dependent control element shapes the quality of the type I IFN response. IFNγ-induced genes either contain GAS or ISRE/IRF elements, irrespective of the presence of CHX. The enrichment of ISRE/IRF containing promoters, even in the absence of translation, is consistent with a direct role of STAT2 in IFNγ signalling (8,9,62). Consistently, a novel subset of genes induced by IFNγ containing ISRE and κB motifs within their promoters and critically requiring the inhibitor of κB kinase beta (IKKβ) has recently been described (63).

Apart from JAK-STAT pathways, IFNs initiate a variety of other signal transduction modules, e.g. mitogen-activated protein kinase, phosphatidylinositol-3-kinase, calcium-calmodulin-dependent protein kinase II, PK-C, cJUN and others (64,65). With the sole exception of transcription factors recognizing DNA motifs overlapping with ISRE/IRF or GAS sites (e.g. AREB6), which we consider to mainly constitute ‘hitchhikers’, we did not observe any other known DNA-binding motifs of other transcription factors included in the TRANSFAC database or random hexanucleotide sequences to be significantly enriched among IFN-induced genes (data not shown). Only STAT and IRF proteins left an obvious and significant footprint during the first hour of IFN treatment. Attempting to identify further enriched elements, we conducted a motif discovery approach (MEME), which yielded only one additional significantly enriched motif, which consists of three GAAA stretches (data not shown). A similar extended ISRE element (containing three AAA elements—two of them GAAA) was recently shown to mediate the IRF-1-dependent TRIM22 induction by IFNγ (66). Additionally, independent IRF-1 ChIP-seq studies revealed the importance of this motif (67). This total dependence of IFN responses on STAT and IRF proteins is consistent with the total absence of ISG expression and antiviral activity of IFNα and IFNγ in cells derived from STAT1-deficient mice (26,68).

The role of IFNγ in transcriptional repression is only partially understood. This is due to the insufficient temporal resolution and sensitivity of current standard technologies (like microarray studies on total RNA) to identify such negative gene regulation when using total cellular RNA. In contrast, 4sU tagging is especially powerful for unmasking these rapid repressive changes in gene expression. We observed IFNγ-mediated repression to be dependent on STAT1 and subjected to negative feedback mechanisms similar to IFNγ-induced changes. In accordance with our findings in mouse fibroblasts, a strikingly similar IFNγ-dependent repression (CHX-resistant and STAT1-dependent) has been reported for the *COL2A1* gene in human chondrocytes, whose promoter does not harbour GAS sites but GC-rich elements (57), suggesting this type of regulation also to be present in human cells.

The biological role of IFNγ-induced gene repression in terms of anti-pathogenic, anti-proliferative and immune-stimulatory function remains to be determined. Nevertheless, based on the annotations of the IRepGs, it is tempting to speculate on potential implications of their repression. It is well-described that several components of IFN induction and signalling are themselves IFN-inducible (see earlier in the text). Conversely, several of the IRepGs (e.g. *Dusp1*, *Dusp10*, *Hes1*) have been previously shown to constitute key negative regulators of innate immune responses and cytokine induction like p38 activation, IL-6 induction and Jak-STAT signalling (69–74). Their repression may thus aid and modulate the establishment of a transient state of hyper alertness for innate immunity related stimuli. Most of the IRepGs encoded transcripts are rather short-lived (median t_½_ = 90 min). This is highly characteristic for genes with key regulatory functions, e.g. transcription factors and genes involved in cell signalling (75). IRepGs are thus likely to represent important regulators of the innate immune response. As we observed repression of IRepGs in both murine fibroblasts and primary macrophages, their regulation represents a core component of the primary IFNγ response.

Interestingly, expression of more than half of the IRepGs showed a strong dependency on STAT1 in IFN-naïve cells. Even though these results were obtained from two different fibroblast cell lines, i.e. NIH-3T3 fibroblasts and immortal STAT1^−^^/^^−^ fibroblasts, it may indicate a dual role of STAT1 in mediating both rapid stimulation of IFN-inducible genes by STAT1-homodimers and maintaining basal expression levels of other genes, presumably constituted by different STAT1-protein complexes. In this respect, it is tempting to speculate that the IFNγ-mediated repression is due to a deprivation of these alternative complexes of STAT1 by the robust IFNγ-mediated STAT1 phosphorylation. It has recently been established that STAT1 dimers change their conformation on IFNγ-induced activation from an antiparallel to a parallel state (76,77), which might instruct such deprivation processes. How this correlates to the overrepresentation of GC-rich elements and putative SP1/SP3 binding sites in their proximal promoter regions remains to be elucidated. Further studies are required to clarify this interesting observation.

As expected, 75 min of CHX treatment resulted in substantial alterations in gene expression. Transcriptional repression was more frequent than induction, which reflects rapid decay of short-lived transcription factor proteins or their regulatory proteins during translational arrest. Within the CHX-induced genes, we detected significant overrepresentation of NF-κB sites consistent with preferential loss of the rather short-lived IκBα protein and subsequent activation of NF-κB signalling. CHX-repressed genes were characterized by an enrichment of Myc/Max and E2F-binding sites, both of which are known for their short protein half-life. Interestingly, we found that CHX treatment altered transcription rates of many genes implicated in the regulation of TNF signalling and known to be important for cell survival in the presence of TNF. As such, the abundance of *traf1*, *A20/Tnfaip3*, *rel-b*, *c-Flip* and *IκBα* transcripts was induced, whereas *caspase 3*, *traf4* and *sphingosin kinase*, were significantly repressed. TNF is known to induce rapid activation of NF-*κ*B by inducing a phosphorylation-dependent ubiquitination and proteasomal degradation of *IκBα*, which otherwise sequesters p50:p65 heterodimers from transcriptional activity (78,79). In the absence of cellular gene expression, for example in presence of CHX or actinomycin D, TNF turns into a highly potent pro-apoptotic signal, thereby leading to cell death (80,81). Conversely, if TNF is added to cells that are able to induce NF-κB target gene expression, apoptosis is not initiated. Therefore, NF-κB signalling can be considered as a cell-intrinsic monitoring module to check for gene expression competence (82–84). We were surprised to find that CHX-incubated cells upregulate negative NF-κB signalling molecules and downregulate positive regulators of TNF signal transduction. This suggests that similar mechanisms might act during incomplete or transient blockade of translation to prepare for TNF encounter in an attempt to increase chances of survival. In summary, these results highlight the need to restrict the duration of translational arrest to avoid secondary effects on the biological mechanisms under study. However, various viruses induce a host cell shut-off, for example, by interfering with cap-dependent translation. Therefore, the transcriptional responses elicited by IFNs during impairment of translation might also be of biological relevance.

## SUPPLEMENTARY DATA

Supplementary Data are available at NAR Online.

## AVAILABILITY

All microarray data are available at Gene Expression Omnibus (GEO) Series GSE30457.

## FUNDING

SynthSys is a Centre for Integrative Systems Biology (CISB) funded by the Biotechnology and Biological Sciences Research Council (BBSRC) and the Engineering and Physical Research Council [BB/D019621/1]; NHS Blood and Transplant grant [WP11-05] and Medical Research Council fellowship grant [G1002523] (to L.D.). Funding for open access charge: Medical Research Council, UK.

*Conflict of interest statement*. None declared.

## Supplementary Material

Supplementary Data
