# Supplementary material for: Deciphering the modulation of gene expression by type I and II interferons combining 4sU-tagging, translational arrest and in silico promoter analysis
Source: Nucleic Acids Res. 2013 Jul 5;41(17):8107–25. doi: 10.1093/nar/gkt589 (PMC3783172; doi:10.1093/nar/gkt589)
Supplement: Supplementary Data [file supp_41_17_8107__index.html]

Deciphering the modulation of gene expression by type I and II interferons combining 4sU-tagging, translational arrest and in silico promoter analysis — Deciphering the modulation of gene expression by type I and II interferons combining 4sU-tagging, translational arrest and in silico promoter analysis — Supplementary Data 

# Deciphering the modulation of gene expression by type I and II interferons combining 4sU-tagging, translational arrest and *in silico* promoter analysis

## 

files

**Files in this Data Supplement:**

- Supplementary Data - xls file
- Supplementary Data - xls file
- Supplementary Data - xlsx file
- Supplementary Data - xlsx file
- Supplementary Data - xls file
- Supplementary Data - doc file
