# Supplementary material for: Deciphering the modulation of gene expression by type I and II interferons combining 4sU-tagging, translational arrest and in silico promoter analysis
Source: Nucleic Acids Res. 2013 Jul 5;41(17):8107–25. doi: 10.1093/nar/gkt589 (PMC3783172; doi:10.1093/nar/gkt589)
Supplement: Supplementary Data [file supp_gkt589_nar-00950-v-2013-File014.doc]

**Supplementary Figure Legends**

**Coupling of 4sU-tagging, Translational Arrest and *in silico* Analysis Deciphers Gene Modulation by IFNα and IFNγ**

Trilling *et al*., 2011

**Supplementary Figure 1**

**Schematic representation of the bioinformatics pipeline**

Microarray data

Sequence logos

Selected

gene set

Quality assessment

gc-RMA

normalization

Intensity filtering

Empirical Bayes

moderated t-test

& BH correction

Non-redundant &

non-overlapping

promoter datasets

Background

gene set

TFBS predictions with

vertebrate PWMs

Mann-Whitney-Wilcoxon

test for each PWM

PWM selection:

Enrichment, proportion of

promoters with sites and

BH corrected p-values

Combine gene lists

(Dölken et al. RNA 2008)

gc-RMA: robust multiarray averaging; BH: Benjamini and Hochberg; TFBS: transcription factor binding site; PWM: position weight matrix.

**Supplementary Figure 2**

**Effect of Cycloheximide on canonical histone mRNA synthesis, processing and decay**

The dramatic ‘induction’ of canonical histones by CHX treatment detectable in nascent RNA was surprising and specific to canonical histones, i.e. not seen for non-canonical histones like histone H2A.Z and H3.3. In contrast to the canonical histones, non-canonical histones posses a 3’-polyA tail and are expressed in a cell-cycle independent manner. To dissect the relative contributions of CHX-induced changes in RNA synthesis, processing and decay in this “induction”, we treated NIH-3T3 cells with cycloheximide for 75 min and performed 4sU-tagging for the last 30, 15, 10 and 5 min. To further exclude temporal alterations in histone transcription rates during CHX-treatment we also included 4sU-tagging from 45 to 50 min of CHX-treatment. **(A)** Histone H1c expression was quantified in nascent and total RNA by quantitative RT-PCR. **(B)** The non-canonical histone H2A.Z served as control as this did not show any regulation by CHX in the microarray data. Gapdh was used as a house keeping gene. Combined data of two independent experiments are shown.

Interestingly, we observed a striking dependence of the ‘induction’ of histone H1c on the duration of 4sU-tagging. ‘Induction’ dropped from almost 80-fold down to ~5-fold when the duration of 4sU-tagging was reduced from 30 to 5 min. The same was seen when nascent RNA labelled from 45 to 50 min of CHX treatment was analysed. At the same time total RNA levels of histone H1C had already increased by ~15-fold arguing against a significant contamination of nascent RNA with total RNA. As expected, no effect was observed for histone H2A.Z.

We conclude that the dramatic ‘induction’ of canonical histone mRNAs by CHX-treatment detectable in nascent is predominantly due to changes in histone mRNA processing which is consistent with previous studies (Sittman et al., 1983; Stimac et al., 1984; Whitfield et al., 2004).


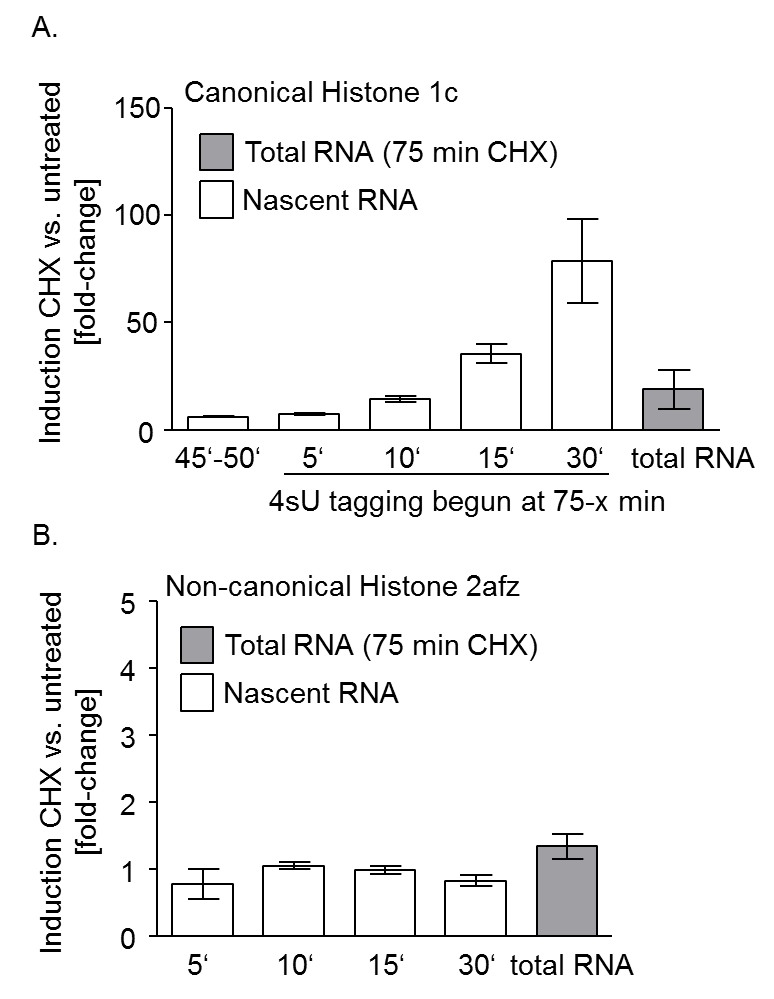


**Supplementary Figure 3**

**Co-operative nature of transcription factor binding site enrichment in relation to transcriptional changes for genes containing only ISRE or only GAS sites in their proximal promoter regions.**

To exclude that regulation via ISRE/IRF sites might simply mask co-operative effects of GAS sites (or *vice versa)*, we had a closer look at IFNα- (A) or IFNγ- (B) inducible genes which lack ISRE/IRF sites but harbour no, at least one or more than one GAS site within their promoter/enhancer elements (or *vice versa*). The median fold induction (depicted in a log_2_-scale) of IFNα- and IFNγ-induced genes is shown in respect to the number of ISRE/IRF or GAS elements in their promoter/enhancer (0, 1 or >1) upon IFN incubation in absence (upper panel) or presence (lower panel) of CHX. The p-value of the correlation between number of sites and induction is indicated in the diagrams. Interestingly, we found that GAS sites seemingly act co-operative upon IFNγ and CHX co-treatment but not upon IFNγ-treatment alone which might indicate that negative feed-back-loops mask such additive effects on promoters containing multiple GAS sites.

**Supplementary Figure 4**

**Measuring the response of NIH-3T3 and STAT1^-/-^ cells to IFNγ using nCounter technology**

NIH-3T3 and STAT1^-/-^ fibroblasts were treated with 100 U/ml of IFNγ or mock for 60 min. 500 µmol 4sU was added from 30 to 60 min of treatment. Newly transcribed RNA was purified and subjected to nCounter measurements for transcripts of 50 selected genes. Data of two independent experiments consisting of two biological replicates each are shown. Data were normalised for the seven house-keeping genes. Shown here is the effect of IFNγ treatment on 12 IFNγ-inducible genes (positive control) and on 7 additional genes involved in IFN signalling (controls).

**
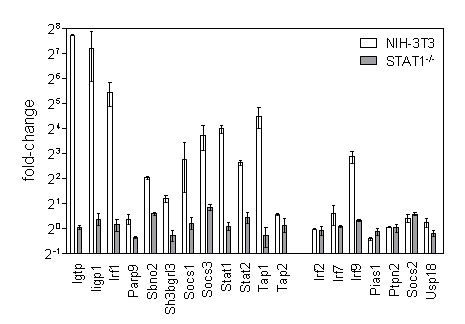
**
